# Pupillary dilation response reflects surprising moments in music.

**DOI:** 10.16910/jemr.11.2.13

**Published:** 2018-12-14

**Authors:** Hsin-I Liao, Makoto Yoneya, Makio Kashino, Shigeto Furukawa

**Affiliations:** NTT Communication Science Laboratories, NTT Cooperation,, Japan

**Keywords:** Pupil, music, surprise, salience, decision making, familiarity, eye tracking, attention, art perception, individual differences

## Abstract

There are indications that the pupillary dilation response (PDR) reflects surprising moments in an auditory sequence such as the appearance of a deviant noise against repetitively presented pure tones (4), and salient and loud sounds that are evaluated by human paricipants subjectively (12). In the current study, we further examined whether the reflection of PDR in auditory surprise can be accumulated and revealed in complex and yet structured auditory stimuli, i.e., music, and when the surprise is defined subjectively. Participants listened to 15 excerpts of music while their pupillary responses were recorded. In the surprise-rating session, participants rated how surprising an instance in the excerpt was, i.e., rich in variation versus monotonous, while they listened to it. In the passive-listening session, they listened to the same 15 excerpts again but were not involved in any task. The pupil diameter data obtained from both sessions were time-aligned to the rating data obtained from the surprise-rating session. Results showed that in both sessions, mean pupil diameter was larger at moments rated more surprising than unsurprising. The result suggests that the PDR reflects surprise in music automatically.

## Introduction

“The eyes are the windows to the soul”—by looking into a person’s eyes, we may understand how she or he thinks and feels. Scientists have backed up this proverb by showing that the pupil reflects various cognitive functions such as cognitive processing load (1, 2, 3), emotion (5), attentional modulation (6, 7), memory (8, 9), decision making (10, 11), high level visual content information processing (13), and mental imagery (14). The underlying mechanism is considered to be related to the locus coeruleus (LC)–norepinephrine (NE) function, which modulates adaptive gain and optimizes performance (15). Since changes in pupil size are tightly coupled with the activity of the LC neurons, we may infer the LC-NE function by observing pupillary responses.

The auditory system is sensitive to stimulus regularity and detects any change rapidly to optimize environmental monitoring. It has been demonstrated that pupillary responses reflect salient and surprising auditory events (e.g., 4, 12, 16, 17, 18, 19, 20). For example, Liao, Yoneya, et al. (4) showed that when participants listened to an auditory sequence consisting of repetitive tones with a deviant noise oddball presented occasionally, pupil size increased when the oddball appeared. This pupillary dilation response (PDR) was observed regardless of whether the participant paid attention to the auditory sequence or not, suggesting that the PDR is an automatic physiological response for auditory surprise detection.

The PDR reflects a surprising moment not only when the surprise is defined objectively as a deviant oddball event against the background, but also when it is defined by human participants’ subjective evaluations. Liao, Kidani, et al. (12) presented ten discrete environmental sounds to participants while their pupillary responses were recorded. Each sound was presented for 500 ms with a 10-s inter-stimulus interval. After the pupillary response recording, they were asked to rate several psychoacoustic aspects of the sounds, including salience, loudness, preference, beauty, hardness, vigorousness, and annoyance. Results showed that the pupil dilated when the sounds were presented. Most importantly, the magnitude of the PDR was positively correlated with the subjective salience of the sound, as well as its loudness, but not with other aspects of the psychoacoustic judgments.

The correspondence between auditory surprise and the PDR shown in our previous studies was found when the salient auditory event was briefly presented, e.g., 50 ms for the noise oddball (4) and 500 ms for the environmental sound (12). In real-world situations, on the other hand, a salient auditory event may last long and continuously. Therefore, it is important to examine whether the PDR reflects auditory salience in complex auditory scenes. In the current study, we examined whether the PDR reflects subjective auditory surprise in music and how loudness may contribute to the effect. Music is a long-lasting, continuous, complex, and yet structured auditory stimulus. A composition usually consists of certain repetitions and variations of the repetitive structure. These characteristics of music enable us to trace subjective surprise evaluations as an excerpt changes. We examined whether the pupil dilates when an excerpt is evaluated as surprising.

## Methods

Participants listened to an excerpt of music for 90 s and concurrently rated how surprising it was, i.e., rich in variation versus monotonous, by sliding a rating bar continuously. Meanwhile, we had them fixate a central point on the monitor to record their pupillary responses. Each participant listened to 15 excerpts of classical, jazz, and rock music. After the concurrent surprise-rating session, participants listened to the same excerpts again while their pupillary responses were recorded, but they were not involved in any task.

### Participants

Twenty-two adults (aged 22-43; median of 35) participated in the study. All had normal or corrected-to-normal vision and reported normal hearing. All participants were naïve about the purpose of the study and received payment for their participation. All the procedures were approved by the NTT Communication Science Laboratories Ethical Committee, and all participants gave informed written consent before the experiment.

### Materials

Stimuli were generated and controlled by a personal computer (Dell OptiPlex 980) and presented through a headphone (Sennheiser HD 595) and on an 18.1-inch monitor (EIZO FlexScanL685Ex). Auditory stimuli were 15 excerpts of the first 90 s of selected pieces (Table 1). They were selected because their structure consisted of both several repetitions and variations of them. The sound pressure levels were fixed across the participants at a comfortable listening level. The visual stimulus was a dark gray fixation point (0.25 × 0.25°, 0.33 cd/m^2^) presented against light gray background (27.0 cd/m^2^).

**Table 1.** Excerpts used in the current study. Artists are indicated with italics.

	Classical	Jazz	Rock
1	Beethoven: Symphony #5 In C Minor, Op. 67, "Fate": Allegro Con Brio *Carlos Kleiber; Vienna Philharmonic Orchestra*	Autumn Leaves *Cannonball Adderley*	(I Can't Get No) Satisfaction *The Rolling Stones*
2	Bach: Chorale "Jesus bleibet meine Freude" *Orchestre de Chambre de Jean-Francois Paillard*	Somethin' Else *Cannonball Adderley*	London Calling *The Clash*
3	Mozart: Serenade No.13 in G major, K.525, "Eine kleine Nachtmusik" Allegro *I Musici*	Blue Train *John Coltrane*	Smells Like Teen Spirit *Nirvana*
4	Chopin: Nocturnes: No. 2 In E Flat Op. 9 No. 2 *Yundi Li*	Moanin *Art Blakey and the Jazz Messengers*	Comfortably Numb *Pink Floyd*
5	Stravinsky: Pétrouchka. Scenes De Ballet, Russian Dance *Phillip Moll: Piano / Berlin Philharmonic Orchestra: Cond: Bernard Haitink*	Waltz for Debby *Bill Evans*	Highway Star *Deep Purple*

Behavioral responses were collected from a transducer (TSD115) connected to a Biopac MP system (HLT100C module, BIOPAC Systems, Inc.). The transducer had a slider on the panel to allow participants to report subjective assessments from 0 to 10 continuously. The sampling rate of the transducer was 1000 Hz. Pupillary responses were recoded binocularly by an infrared eye-tracker camera (Eyelink 1000 Desktop Mount, SR Research Ltd.) with a sampling rate of 1000 Hz.

### Design

The 15 musical excerpts were presented twice in different sessions: first in a surprise-rating session and then again in a passive-listening session. The order of the excerpts in each session for each participant was randomly assigned. The inter-stimulus interval (ISI) was 5 s. The total duration of each session was around 25 min.

### Procedure

All participants were given written and oral explanations about the nature of the experiment and the pupillary response recording. Participants sat in front of the monitor at a viewing distance of 51 cm in a dimly lit soundproof chamber, with their chin on a chinrest. Before each session, a five-point calibration procedure was performed, after which the participants were instructed to fixate the central point throughout the experiment.

In the surprise-rating session, participants were asked to concurrently rate how they felt about changes (in any sense) compared with the portions within the excerpt they had heard so far. For example, if they felt any aspect in the music, including melody, tempo, harmony, or texture (e.g., more instruments playing), became richer in variation, they moved the slider to the right to register higher scores. If they felt the change became monotonous, they moved it to the left to register lower scores. The slider was reset in the middle (i.e., scored as 5) at the beginning of each excerpt.

In the passive-listening session, participants listened to the same musical excerpts again without any task involvement. The break between the two sessions was longer than 30 min. The order of the two sessions was fixed to avoid the influence of expectation on the surprise rating due to the repetition.

After the two sessions, participants answered a questionnaire to rate from 1 (never heard the piece) to 7 (often heard the piece) how familiar they were with each excerpt and to write down the name of the piece and/or the artist/composer if they knew it. They were allowed to replay the excerpts at their own pace when answering the questionnaire.

## Results

### Familiarity with the excerpts (questionnaire)

The mean familiarity scores for the classical, jazz, and rock music were 4.1, 2.4, and 2.6, respectively (scores for individual excerpts are listed in Table 2). The mean scores for each participant were subjected to a repeated-measures ANOVA with the music genres (classical, jazz, rock) as within-subject factors. Results showed that participants were more familiar with the classical music we selected than the other types of music [*F*(2,42) = 19.07, *p* < .001, *η^2^* = .48]. The results of the open questions are shown in Table 2 (second and third columns). Participants tended to give more answers and correct ones to questions about the classical music than to those about the other genres, which is consistent with the results of the subjective feeling of familiarity.

**Table 2.** Results of familiarity rating, questionnaire, and on-line surprise rating. The first column shows the means of the familiarity rating score across participants, with the standard deviation in parentheses. The second and third columns show the number of participants who gave any answer and a correct one to questions about the excerpt or the artist/composer, respectively. The fourth and fifth columns show the mean of the average and standard deviations of the surprise rating over time, respectively, across participants. Numbers in parentheses are standard deviations across participants. The last column shows the Kendall’s coefficient of concordance (W) of the on-line surprise rating.

		Familiarity rating	Total answers	Correct answers	Average surprise rating over time	Variations in surprise rating over time	Kendall’s W
*Classical*	1	5.0 (1.7)	17	16	6.4 (1.1)	1.4 (0.6)	0.39
	2	4.4 (1.8)	8	4	4.9 (1.3)	1.1 (0.8)	0.56
	3	5.0 (1.3)	7	5	6.2 (1.0)	1.4 (0.6)	0.30
	4	4.6 (1.7)	10	9	5.0 (1.8)	1.0 (0.5)	0.71
	5	1.7 (1.1)	1	0	5.2 (1.5)	1.3 (0.8)	0.50
*Jazz*	1	1.8 (1.5)	1	1	4.7 (1.3)	1.1 (0.8)	0.52
	2	1.6 (1.4)	0	0	5.1 (2.4)	1.2 (1.0)	0.66
	3	2.5 (1.8)	0	0	5.2 (1.7)	1.1 (0.5)	0.63
	4	3.5 (1.7)	0	0	5.7 (1.3)	1.2 (0.7)	0.47
	5	2.4 (1.6)	0	0	5.5 (1.8)	1.0 (0.4)	0.73
*Rock*	1	3.4 (2.0)	3	3	6.2 (1.4)	1.0 (0.6)	0.64
	2	1.5 (1.2)	1	1	6.1 (2.1)	1.3 (0.9)	0.62
	3	3.3 (2.3)	4	4	6.4 (1.5)	1.2 (0.6)	0.52
	4	1.6 (1.2)	1	0	4.1 (1.5)	1.1 (0.6)	0.53
	5	3.0 (1.9)	1	1	6.7 (1.8)	1.1 (0.4)	0.70

### On-line subjective surprise rating

**Figure 1.**
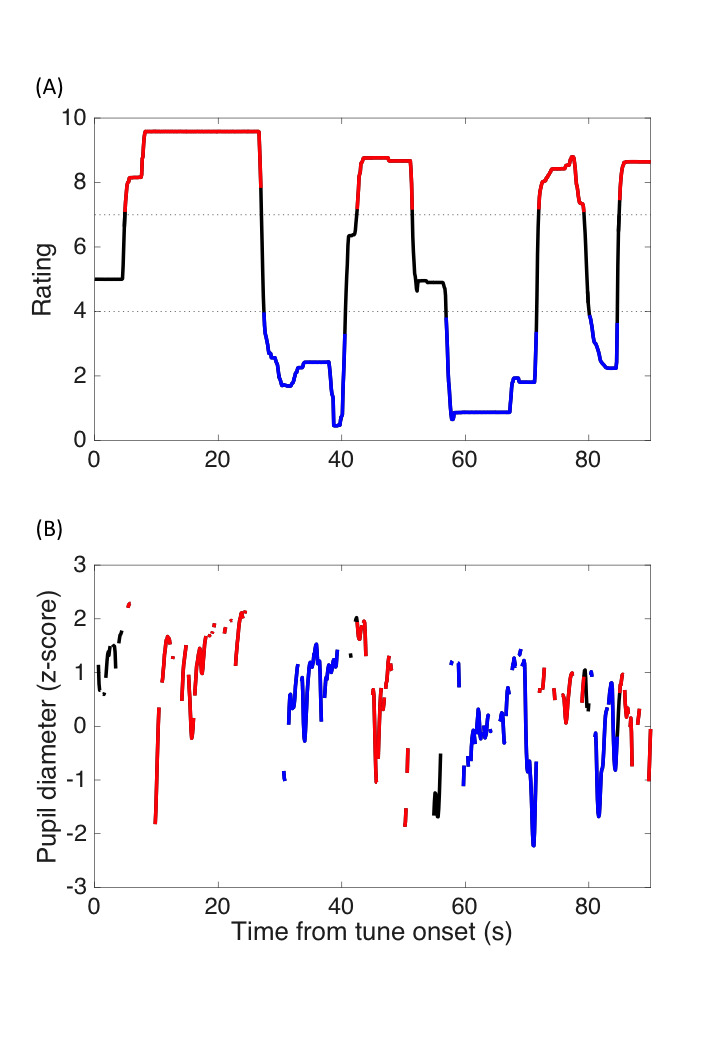
Examples of on-line surprise rating (A) and pupil size change (B) over time. The red and blue lines represent the surprising and unsurprising moments, respectively, as defined as when the rating score was above 7 or below 4.

Figure 1A shows examples of the surprise rating over time. The average (the fourth column) and variation (the fifth column) of the surprise rating over time are listed in Table 2.

To examine whether the surprise rating varied among the music genres or excerpts (e.g., in terms of its familiarity), we conducted two different analyses. First, the means of the average, as well as the standard deviations, of the surprise rating score were subjected to a repeated-measures ANOVA with the music genres (classical, jazz, rock) as within-subject factors. Results showed that neither the average surprise rating [*F*(2,42) = 2.80, *p* > .07, *η^2^* = .12] nor the variations in the surprise rating over time [*F*(2,42) = 1.13, *p* > .3, *η^2^* = .05] differed among music genres. Second, we calculated the correlation between the familiarity rating and average surprise rating and the correlation between the familiarity rating and the variations in the surprise rating over time. Results showed a positive correlation between familiarity and the average surprise rating over time (*r* = .24, *p* < .001) but not between familiarity and variations in the surprise rating over time (*r* = -0.07, *p* > .2).

To examine the consensus among the participants on the surprise rating, we calculated Kendall’s coefficient of concordance (W). The rating data were resampled with a 10-Hz sampling rate for the analysis. The results are shown in Table 2 (sixth column). The consensuses among the participants were moderate but significant, and they varied among musical excerpts (median of 0.56, min of 0.30, max of 0.73; all *p*s < .001).

### Pupillary response analysis

Figure 1B shows the pupil size change over time. Only data recorded from the right eye were analyzed since the pupillary responses from both eyes were consensual. Data during blinks were treated as missing and discarded (30.1%). The range of the average blink rate was about the same as in our previous studies (4, 12), where the task was an auditory one that allowed normal blinks.

The pupil size measurement in the video-based eye tracker system, as used in the current study, was covariant with the gaze position (21). To avoid recording errors due to unexpected gaze positions, pupil size data were screened when the gaze position deviated 1.5 deg. from the central fixation point, and 23.1% of the data were screened out.

The Eyelink system outputs arbitrary units [au] to represent the pupil size, which was not calibrated across participants or conditions. To compare the results across conditions, we computed z-score during each 90-s excerpt. To reduce high-frequency noise due to the over-fine sampling rate (i.e., 1000 Hz) for pupillary response measurements, we resampled the data with a 10-Hz sampling rate for the analysis. Specifically, the data between the resampling points (i.e., every 100 data points) were discarded without any interpolation or filtering procedure. In this work, we used an EDF converter (provided by SR Research) to convert the Eyelink EDF file to the ASC format, and we used Matlab for all the data analyses. The function for the resampling procedure described above was “downsample.” We followed the same protocol as in our previous study (4).

### Surprise-related PDR

The pupil data recorded in the two sessions (surprise-rating and passive-listening) were time-aligned to the rating data obtained in the surprise-rating session. The surprising moments were defined arbitrarily as the period when the surprise rating score was above 7 (the red lines in Fig. 1), the unsurprising moments as a surprise rating score below 4 (the blue lines in Fig. 1), and the neutral moments as a surprise rating score between 7 and 4 (the black lines in Fig. 1). The criterion was set to obtain similar probabilities of the valid data for surprising and unsurprising moments: 24.7% and 21.7% of the total duration, respectively.

**Figure 2.**
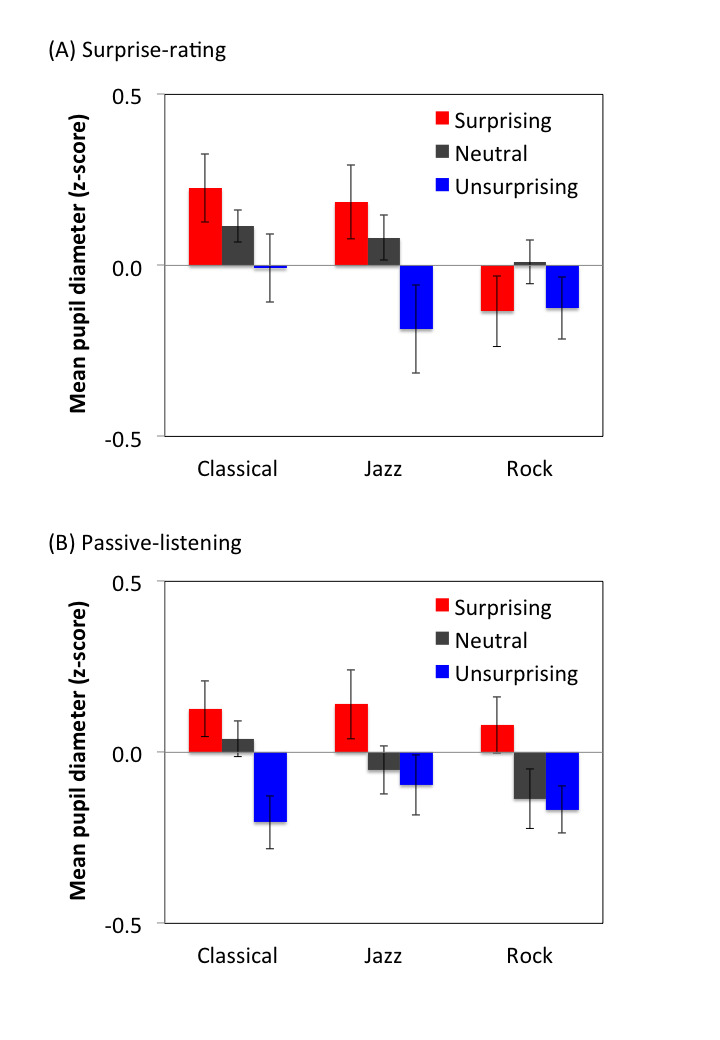
Mean of the pupil diameter during the surprising, neutral, and unsurprising moments parameterized by music type in surprise-rating (A) and passive-listening (B) sessions. Error bars represent standard errors across participants.

Results are shown in Fig. 2. Mean pupil diameter was subjected to a three-way repeated-measures ANOVA with the task (surprise-rating, passive-listening), music genre (classical, jazz, rock), and surprise (surprising, neutral, unsurprising) as within-subject factors. Results showed main effects of surprise [*F*(2,42) = 9.66, *p* < .001, *η^2^* = .32] and music genre [*F*(2,42) = 3.31, *p* < .05, *η^2^* = .14] but not any other effect or interaction (*p*s > .1). When we applied a different criterion to define the surprising moments in which the deviation of the rating score from mean was more than 1.5 times the standard deviation, the effect of surprise remained [*F*(2,42) = 10.82, *p* < .001, *η^2^* = .34]. The results suggest that the pupil dilated more strongly during the surprising moments than during the unsurprising ones regardless of the music genre or whether the on-line surprise rating was involved or not.

**Figure 3.**
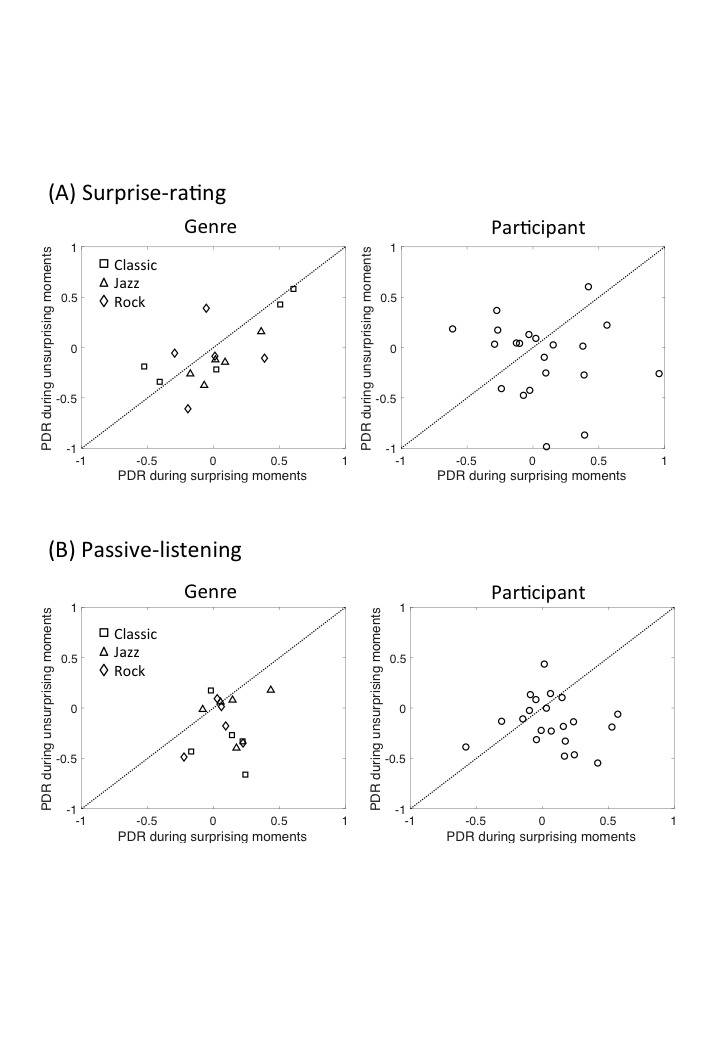
Scatter plots of PDR during the surprising moments against PDR during the unsurprising ones in surprise-rating (A) and passive-listening (B) sessions. Each marker represents each musical genre (in the left panels) or participant (in the right panels).

To further investigate whether there was systematic bias induced by a particular musical excerpt or participant, we used scatter plots to represent the surprise-related PDR for individual excerpts and participants. Results are shown in Fig. 3. The data were clustered below the diagonal line (confirming larger PDR during surprising moments than during unsurprising ones), while the distribution of the genres or participants was spread equally, indicating a consistent tendency of the surprise-related PDR among different genres or participants. There was no significant correlation between the surprise-related PDR (i.e., the difference in average pupil size between surprising moments and unsurprising ones) and the familiarity rating (*r* = .02, *p* > .8 for the surprise-rating condition, and *r* = .03, *p* > .7 for the passive-listening condition; data not shown). The overall results suggest that the surprise-related PDR did not depend on the genre, participant, or familiarity with the excerpt.

**Figure 4.**
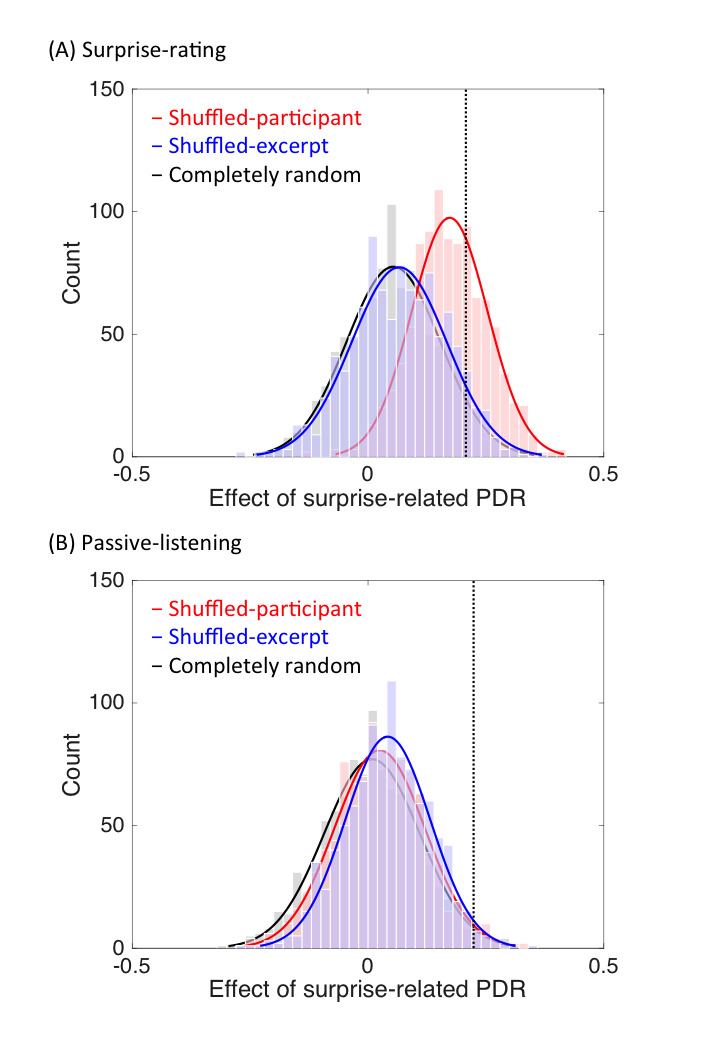
Histogram of the estimated PDR-surprise associations in surprise-rating (A) and passive-listening (B) sessions. The curves represent the fitted normal densities. The vertical dashed lines indicate the observed surprise-related PDR.

We conduced further analysis to verify the effect of the surprise-related PDRs and examine whether the effect could be explained by stimulus-driven factors coupled with the musical excerpts or response biases/tendencies associated with the participants. Specifically, we calculated the estimated PDR-surprise association using bootstrapping procedures. In the completely random procedure (as a baseline), the pupil data were aligned with rating data randomly selected from different participants/excerpts. The difference in the mean pupil diameter between surprising and unsurprising moments, derived from the ratings of different participants and excerpts, was calculated 1,000 times (by random selection between the pair of the pupil and rating data) to form a distribution, where the PDR was expected not to be associated with the surprise at all. The results are shown in Fig. 4. In both the surprise-rating and passive-listening conditions, the baseline distributions (i.e., the black distributions) were quite distant from the observed surprise-related PDR (indicated as vertical dashed lines), indicating a reliable surprise-related PDR: when the pupil data matched the rating data for the same participant and musical excerpt, pupil size was larger during surprising moments.

We further calculated the estimated PDR-surprise associations when the pupil data were paired with the rating data for the same excerpt, but randomly selected from different participants (i.e., shuffled-participant condition), and when the pupil data were paired with the rating data obtained from the same participant, but randomly selected from different excerpts (i.e., shuffled-excerpt condition). We expected that if the observed surprise-related PDR could mainly be explained by the stimulus-driven factor, the distribution of the estimated PDR-surprise association from the shuffled-participant condition would be close to the observed surprise-related PDR. Namely, as long as the pupil data were aligned with the rating data from the same excerpt, regardless of the rater/participant, the PDR-surprise association would increase. In contrast, if the surprise-related PDR could mainly be explained by the participant-related factors, such as response bias systematic tendency of rating, etc., the surprise-related PDR would be close to the estimation obtained from the shuffled-excerpt procedure. Namely, the surprise-related PDR would be due to coordination between the pupillary response and rating of a particular rater/participant, regardless of the excerpt that was to be rated.

As shown in Fig. 4, in the surprise-rating session, the observed surprise-related PDR was quite close to the distribution derived from the shuffled-participant procedure but not to the distribution derived from the shuffled-excerpt procedure, indicating that the stimulus characteristics might contribute to the surprise-related PDRs during surprise rating. In contrast, no such result was found in the passive-listening session. The distribution of the shuffled-participant or shuffled-excerpt condition overlapped the baseline distribution and was distant from the observed surprise-related PDR. The results suggest that the surprise-related PDRs observed during passive listening cannot be explained by the stimulus characteristics or response biases/tendencies associated with the participants.

### Decision-making-related PDR

Surprise-related PDRs were observed in both the surprise-rating and passive-listening sessions. It may be suspected that the participant performed the surprise rating implicitly and spontaneously even though they were not asked to, especially since the passive-listening condition was always conducted in the later session. To examine whether the participant performed the rating while listening to the music, we conducted another analysis to examine the decision-making-related PDR (e.g., 10, 11).

**Figure 5.**
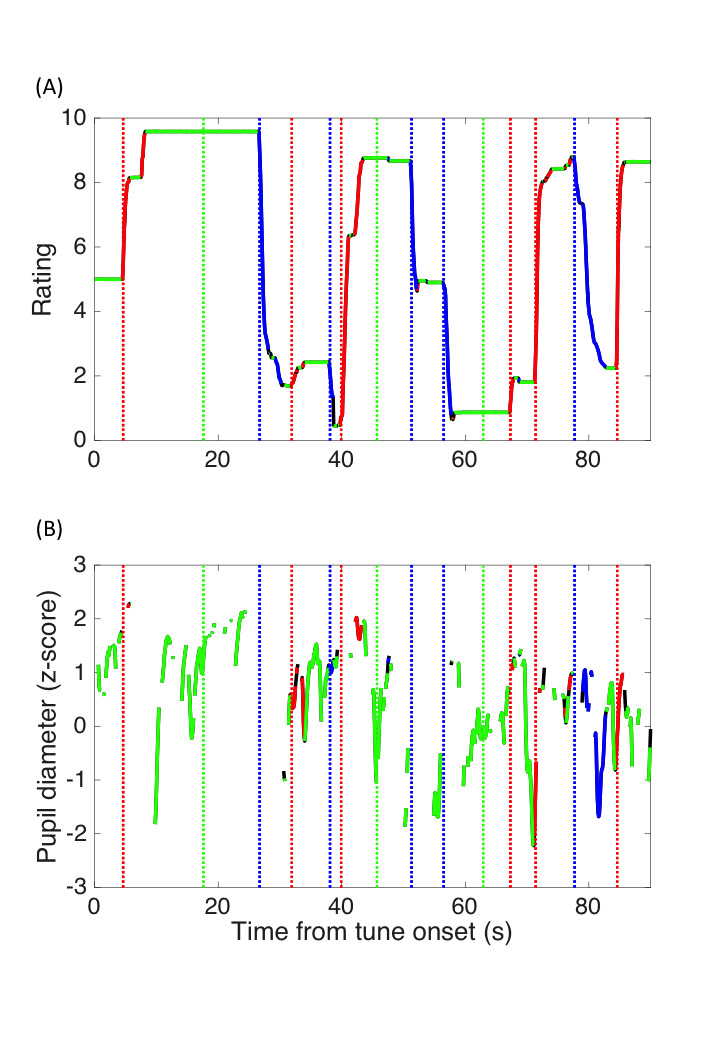
Examples of on-line surprise rating (A) and pupil size change (B) over time. The lines represent the surprising (red), unsurprising (blue), and the unchanged (green) moments, which were defined as the period when the rating score was increased, decreased, or unchanged over time. The vertical dotted lines represent the reference timing of the surprising change (red), unsurprising change (blue), and unchanged (green) rating.

The pupil data were time-locked to the rating change instead of the rating moment as shown in the surprise-related PDR analysis. The rating change was defined as the moment the rating started to move in a particular direction. Figure 5 shows examples of the results of the time-locked analysis of the rating change. The timing of the surprising change was defined as the start of the period in which an increased rating score lasted longer than 0.5 s (the vertical red dotted lines). The timing of the unsurprising change was defined as the start of the period in which a decreased rating score lasted longer than 0.5 s (vertical blue dotted lines). To provide the baseline, we defined the neutral time as the median timing of the period in which an unchanged rating score lasted longer than 4 s (vertical green dotted lines).

**Figure 6.**
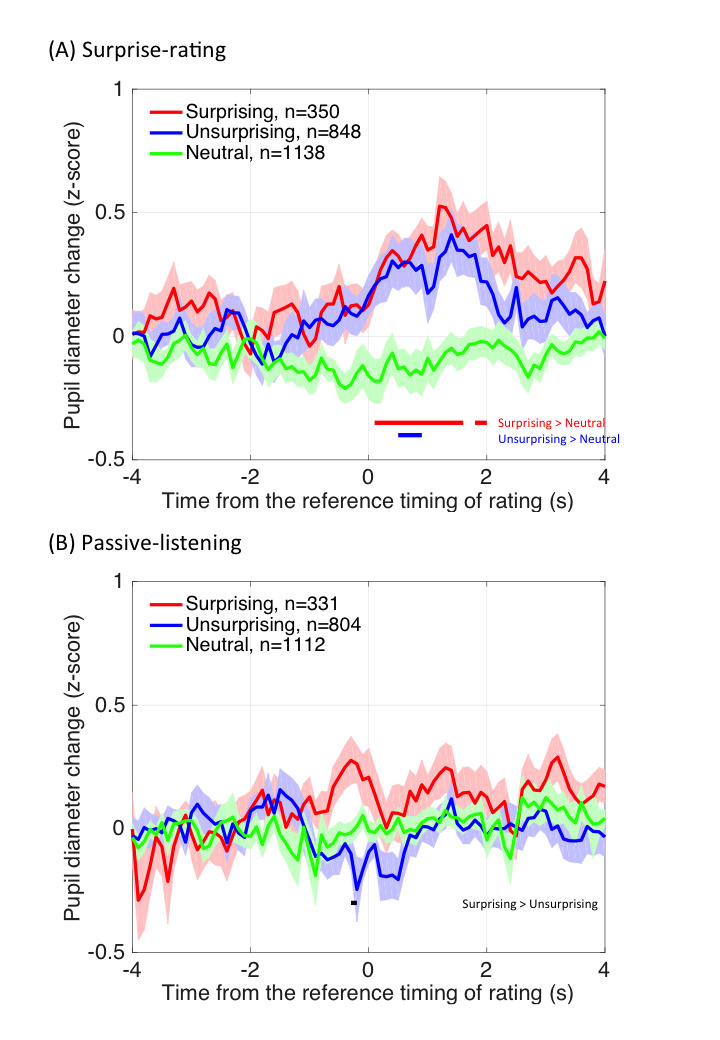
Mean pupil diameter change time-locked to the reference timing in surprise-rating (A) and passive-listening (B) sessions. The shadow represents standard errors across participants. The horizontal lines represent significant differences between surprising and neutral conditions (red lines), unsurprising and neutral conditions (blue lines), or surprising and unsurprising conditions (black lines), p < .05 with Bonferroni correction. Ns represent the number of valid trials from all participants for each condition.

Mean pupil diameter changes time-locked to the reference timing are shown in Fig. 6. To examine whether pupil diameter reliably increased, we conducted a pairwise *t*-test at each time point for all the pairs in the three conditions (Bonferroni corrected *p*-value). Results showed that in the surprise-rating session, pupil diameter increased around 1 s before the decision-making event, and reached statistical significance around the reference timing, as indicated by the difference between the surprising/unsurprising and neutral conditions. Note that the reference timing of the surprising and unsurprising events was when the participant started moving the rating bar. The pupil dilated before the timing, suggesting that this PDR is evoked by a decision-making processing, rather than motor behavior. This is consistent with Einhäuser et al. (10), who showed that the pupil dilated at the moment a choice was made even when the actual motor response occurred thereafter. This decision-making-related PDR had a similar pattern regardless of whether the decision was made in the surprising or unsurprising direction. In contrast, in the passive-listening condition, no such decision-making-related PDR was found. The overall results suggest that there was no spontaneous decision-making process involved in the passive-listening session.

### Loudness-related PDR

We have previously found that the subjective salience evaluation of sounds is highly correlated with their loudness, as well as with the PDR to them (12). The sounds used in the previous study were environmental sounds presented briefly (500-ms duration) and discretely (10-s ISI). It remains unclear whether the current online surprise judgment on the long-lasting music can be explained by the instantaneous loudness change of the music, and whether the surprise-related PDR can be simply explained by the loudness change.

**Figure 7.**
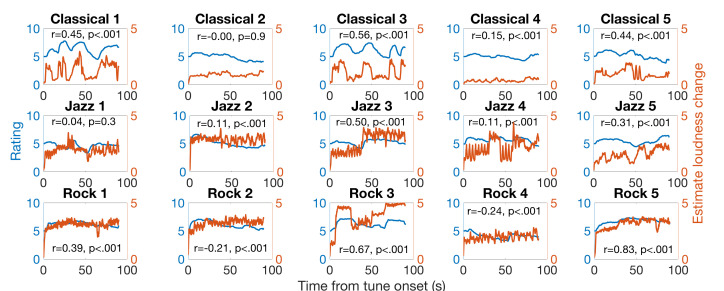
Subjective salience rating (blue lines) and estimated instantaneous loudness change (orange lines) over time. Pearson’s correlation coefficients with hypothesis testing p-values are shown for each excerpt.

To investigate the issue, we conducted an analysis to examine the similarity between the surprise rating and instantaneous loudness change for each excerpt. The loudness of the musical excerpt was estimated by an excitation-pattern-based loudness model (e.g., 22). The acoustic signal was bandpass filtered with a bank of filters of equivalent rectangular bandwidth (ERB) with the center frequencies spaced 0.5 ERB from 30 to 16,000 Hz, and weighted with the middle ear transfer function. The outputs were divided into segments of 100-ms windows to compute the instantaneous loudness. The instantaneous loudness was smoothed with a 1-s window to represent the estimated loudness change over time. We then calculated the correlation between the surprise rating and loudness change over time. Results showed that among the 15 excerpts we selected, 13 showed significant correlation between the surprise rating and instantaneous loudness change (see Fig. 7).

**Figure 8.**
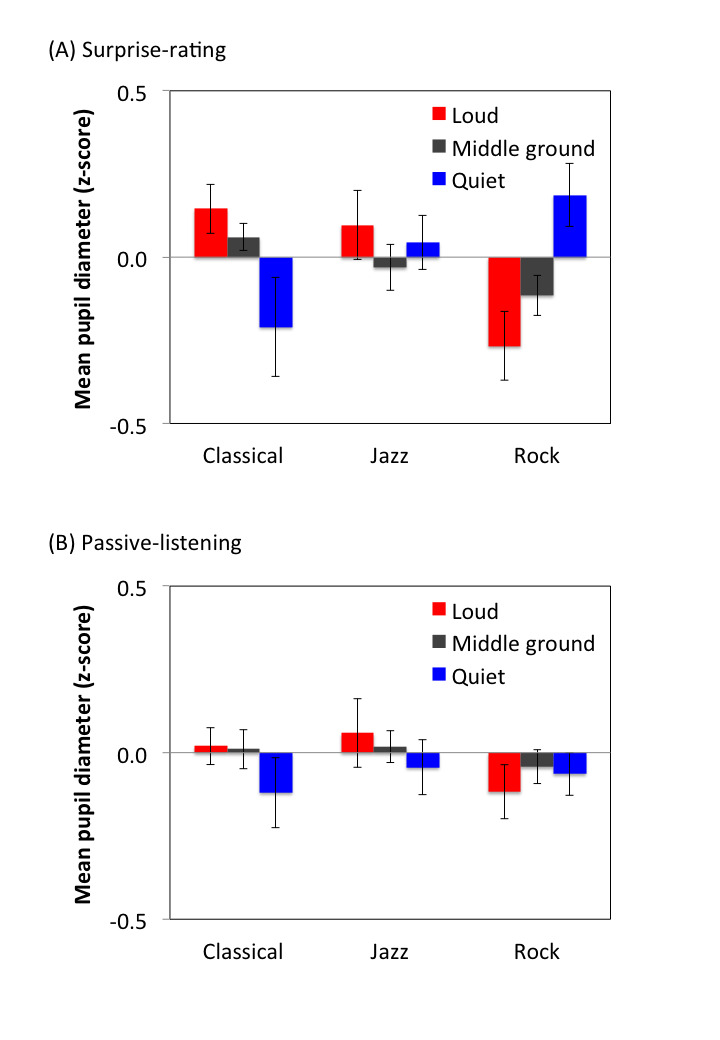
Mean of the pupil diameter during loud, middle ground, and quiet moments parameterized by music type in surprise-rating (A) and passive-listening (B) sessions. Error bars represent standard errors across participants.

To further investigate whether the pupil data simply reflected the instantaneous loudness change of the music, we conducted an analysis of the loudness-related PDR. The idea was to align the pupil data with the loudness change over time, as in the analysis of the surprise-related PDR, to examine whether the pupil size was larger during the loud moments than during the quiet ones. The loud and quiet moments were defined as when the deviation of the loudness change from the mean was larger and smaller than 1.5 times the standard deviation, respectively, and the middle ground between the criteria. Mean pupil diameter during loud, quiet, and middle ground moments (Fig. 8) was subjected to a three-way repeated-measures ANOVA with the task (surprise-rating, passive-listening), music genre (classical, jazz, rock), and loudness (loud, middle ground, quiet) as within-subject factors. Results showed an two-way interaction between music genre and loudness [*F*(4,84) = 7.36, *p* < .001, *η^2^* = .26] and the three-way interaction among task, music genre, and loudness [*F*(4,84) = 2.95, *p* < .03, *η^2^* = .12], but not any other main effect or interaction (*p*s > .3).

## Discussion

We examined whether the PDR reflects surprising moments in music. Participants evaluated how surprisingly a musical excerpt changed over time while they listened to the music concurrently. We found that their pupil size increased at the moment they gave a surprise rating, indicating a surprise-related PDR in music. This pattern of results was also observed when they listened to the music passively without performing any evaluation. Note that in the current study, the surprise-related PDR was not revealed as a typical phasic (or biphasic) response as it is when the surprise event is clearly defined and presented discretely (e.g., 4, 12). In contrast, the ‘surprise’ was defined by a continuously updated processing over time (therefore, it was not necessarily a discrete transient event), as indexed when the surprise rating scores increased beyond certain levels, which might make the stereotypical phasic response less noticeable. In any case, when averaging the pupil size across time periods during ‘surprise’ events, it has been consistently observed that the average pupil size is larger around the surprise events than for background neutral sounds (4) or less surprising/salient sounds (12).

Further bootstrapping analysis demonstrated that the effect of stimulus characteristics might contribute to the surprise-related PDRs during the surprise rating task but not during passive listening. Moreover, the decision-making-related PDRs were only observed when the participants performed the rating task but not when they listened to the music passively, indicating the absence of spontaneous evaluation in the latter case. The overall results indicate that PDR reflects surprising moments in music, regardless of whether an evaluation of the surprise per se is required. This suggests that the surprise-related PDR could be due to a stimulus-driven response to the acoustic features embedded in the music or due to automatic monitoring of surprise in an auditory environment.

The surprise-related PDR was observed for all the music genres we tested, regardless of the familiarity with the excerpt. In the behavioral subjective rating, participants were more familiar with a particular genre of music, i.e., classical music, than the others, and tended to give a higher surprise rating on average over time if they were familiar with the excerpt. The reason for this tendency could be that when one is familiar with a particular excerpts, it becomes easier for him/her to form an expectation and thus to predict the ‘surprise’ or be predisposed to it. It has been shown that with familiarity with excerpts, chills and emotional responses related to the excerpts increase (e.g., 23, 24, 25). Chills are also observed in the reflection of pupillary dilation response (26) and are often present when music is rich in variation. While we did not measure chills or perform an emotional evaluation of the excerpts, it is unclear whether the surprise rating was similar to chills or not. However, the surprise-related PDR did not correlate with familiarity with the excerpts and was observed robustly and constantly regardless of music genre. This suggests that the surprise-related PDR can hardly be explained by familiarity or chills and is consistent with the idea that the reflection of the PDR in auditory surprise is an automatic physiological response. This conclusion is also supported by evidence showing that the PDR to a deviant auditory oddball (4) is independent of the task demand, i.e., when the participant does not pay attention to the oddball per se.

It remains unclear whether and how the subjective surprise evaluation in music can be derived from stimulus-driven factors. The consensus on the surprise rating among the participants was generally at the intermediate level and varied among the musical excerpts, indicating that the evaluation was based on an interaction between the top-down expectation (e.g., knowledge and familiarity with the excerpts) and stimulus-driven factors (e.g., acoustic features). This conclusion is also supported by the results of the bootstrapping analysis of the estimate PDR-surprise associations in that the surprise-related PDR could be explained, but only partly, by the stimulus-driven effects associated with the musical excerpts. Huang and Elhilali (17) investigated auditory salience using natural soundscapes. They asked participants to rate relative salience between two auditory streams and took a data-driven approach to uncover the critical parameters for auditory salience. They found that auditory salience is spaced among multidimensional features that combine nonlinearly and context dependently. Estimating auditory surprise in music requires, in addition to the features contributing to auditory salience, parameters that are possibly related to the time sequence and interactions among the acoustic features to estimate the surprise derived from the passing sequence. A related study has shown how surprise in popular music contributes to preference (27). Considering that pupil size also reflects emotional arousal (28) that might be related to preference, more study is required to further investigate how the pupil reflects surprise and preference and their interaction.

The surprise-related PDR in music cannot be explained by an explicit or spontaneous surprise evaluation of the music or cognitive processing load (in terms of task demand). Einhäuser and colleagues (10) showed that the pupil dilates at the moment a decision is made, regardless of the decision content or whether the motor response is required. This is consistent with our observation of the decision-making-related PDR only in the surprise-rating condition, regardless of the surprising or unsurprising rating, but not in the passive-listening condition. In contrast, surprise-related PDRs were constantly observed in both conditions, and thus cannot be explained by the decision-making process. With regard to the cognitive processing load, the pupil dilates when the load increases (1, 2, 3). In the current study, the task demand was constantly required during the surprise-rating session but not required at all during the passive-listening one, but the surprise-related PDR was constantly observed regardless of whether the cognitive effort was involved or not.

The surprise-related PDR might be partially explained by the loudness change of the music, depending on the music genre. It has been shown that subjective salience evaluation is highly correlated with loudness, regardless of whether the sound is presented briefly (12) or if it is long-lasting music as in the current study. However, the loudness change could not explain the pupillary response to all the music genres. In the loudness-related PDR analysis, larger pupil size during loud moments than during quiet ones was only observed for the classical music, and the effect was more remarkable during the surprise-rating condition than the passive-listening condition. This general pattern is different from the surprise-related PDR, in that the effect was observed for all the music genre. Furthermore, while the consensus of the surprise rating among the participants was at the intermediate level, it is possible that the pupillary response for individual participant did not simply reflect the loudness change, but instead was modulated by each participant’s specific judgment of surprise. The overall results suggest that loudness may partially explain the surprise-related PDR.

Pupillometry has recently been widely used to study various aspects of musical processing such as arousal and preference (29), chills (26), and familiarity (30). The current study contributes to our understanding of pupillary response related cognitive processing by demonstrating that not only emotional arousal induced by music, but also the orienting response by surprise can be revealed by the pupillary response. By presenting relatively long musical excerpts, we were able to apply various analyses to investigate the dynamics of pupillary responses and related cognitive processing. We conclude that the pupil dilates automatically during surprising moments in music.

## Ethics and Conflict of Interest

The authors declare that the contents of the article are in agreement with the ethics described in http://biblio.unibe.ch/portale/elibrary/BOP/jemr/ethics.html and that there is no conflict of interest regarding the publication of this paper.
